# PKC-ζ Aggravates Doxorubicin-Induced Cardiotoxicity by Inhibiting Wnt/β-Catenin Signaling

**DOI:** 10.3389/fphar.2022.798436

**Published:** 2022-02-14

**Authors:** Yan-Jun Cao, Jing-Yan Li, Pan-Xia Wang, Zhi-Rong Lin, Wen-Jing Yu, Ji-Guo Zhang, Jing Lu, Pei-Qing Liu

**Affiliations:** ^1^ National and Local United Engineering Lab of Druggability and New Drugs Evaluation, School of Pharmaceutical Sciences, Sun Yat-sen University, Guangzhou, China; ^2^ School of Pharmaceutical Science, International Institute for Translational Chinese Medicine, Guangzhou University of Chinese Medicine, Guangzhou, China; ^3^ School of Pharmaceutical Sciences, Shandong Academy of Medical Sciences, Shandong First Medical University, Taian, China

**Keywords:** PKC-ζ, doxorubicin, cardiotoxicity, apoptosis, Wnt/β-catenin

## Abstract

Doxorubicin (Dox) is a chemotherapeutic drug used to treat a wide range of cancers, but its clinical application is limited due to its cardiotoxicity. Protein kinase C-ζ (PKC-ζ) is a serine/threonine kinase belonging to atypical protein kinase C (PKC) subfamily, and is activated by its phosphorylation. We and others have reported that PKC-ζ induced cardiac hypertrophy by activating the inflammatory signaling pathway. This study focused on whether PKC-ζ played an important role in Dox-induced cardiotoxicity. We found that PKC-ζ phosphorylation was increased by Dox treatment *in vivo* and *in vitro*. PKC-ζ overexpression exacerbated Dox-induced cardiotoxicity. Conversely, knockdown of PKC-ζ by siRNA relieved Dox-induced cardiotoxicity. Similar results were observed when PKC-ζ enzyme activity was inhibited by its pseudosubstrate inhibitor, Myristoylated. PKC-ζ interacted with β-catenin and inhibited Wnt/β-catenin signaling pathway. Activation of Wnt/β-catenin signaling by LiCl protected against Dox-induced cardiotoxicity. The Wnt/β-catenin inhibitor XAV-939 aggravated Dox-caused decline of β-catenin and cardiomyocyte apoptosis and mitochondrial damage. Moreover, activation of Wnt/β-catenin suppressed aggravation of Dox-induced cardiotoxicity due to PKC-ζ overexpression. Taken together, our study revealed that inhibition of PKC-ζ activity was a potential cardioprotective approach to preventing Dox-induced cardiac injury.

## Introduction

Doxorubicin (Dox) is a widely used chemotherapeutic agent for treatment of leukemia, lymphoma, neuroblastoma, and other human cancers (Carvalho et al., 2014). However, the serious cardiac side effects of Dox, including cardiomyopathy, arrhythmia, and congestive heart failure, limit its cumulative therapeutic dose in clinical application (Wallace et al., 2020). These cardiotoxic effects mainly stem from mitochondrial dysfunction (Wenningmann et al., 2019), autophagy (Bartlett et al., 2017), oxidative stress (Songbo et al., 2019), apoptosis (Takemura and Fujiwara, 2007), and impairment of calcium homeostasis (Octavia et al., 2012). Previous findings of our lab have established that Wnt signaling (Hu et al., 2019), mitophagy (Wang et al., 2019), and PARylation (Lu et al., 2019) played important roles in Dox-induced cardiotoxicity. However, management of Dox-induced cardiotoxicity is complicated by its multifactorial nature and complicated pathogenesis. Therefore, further studies are needed to elucidate mechanisms of Dox-induced cardiotoxicity.

Protein kinase C-ζ (PKC-ζ) is a serine/threonine kinase belonging to atypical protein kinase C (PKC) subfamily (Diaz-Meco and Moscat, 2012). PKC-ζ activation does not depend on calcium and diacylglycerol (DAG) but lipids such as ceramide in contrast to the other two subfamilies including conventional and novel PKCs (Steinberg, 2008). Previous studies report that PKC-ζ regulates diverse biological functions such as cell polarity (Suzuki and Ohno, 2006), inflammation (Diaz-Meco and Moscat, 2012), and apoptosis (Garin et al., 2007). The phosphorylation and activity of PKC-ζ is increased in atherosclerosis (Heo et al., 2011), cardiac hypertrophy (Gao et al., 2018), and ischemia/reperfusion (I/R) injury (Strasser et al., 1999). PKC-ζ peptide inhibitor attenuates polymorphonuclear leukocyte-induced cardiac contractile dysfunction after I/R (Phillipson et al., 2005). Tumor necrosis factor-α (TNF-α) activates PKC-ζ and induces apoptosis of endothelial cells (Kim et al., 2012). PKC-ζ knockdown have been reported to reverse oxidized low-density lipoprotein lipotoxicity in cardiomyocytes (Chen et al., 2017). These findings highlight the crucial role of PKC-ζ in cardiovascular diseases, but its effect on Dox-induced cardiotoxicity remains unknown.

Wnt/β-catenin is a highly conserved Wnt signaling pathway branch. As an important transcriptional co-activator, β-catenin protein dynamic balance plays a key role in maintaining cell homeostasis (He and Tang, 2020). Wnt/β-catenin signaling has been extensively studied as a key regulator of pathological and physiological processes in the cardiovascular field (Tzahor, 2007; Deb, 2014). Previous studies report that Wnt/β-catenin promotes early cardiac differentiation, cardiomyocyte proliferation, and angiogenesis (Cohen et al., 2008; Dejana, 2010). β-catenin protein levels decrease in cardiac I/R injury, and overexpression of β-catenin suppresses ROCK1/PTEN signaling pathway and protects against transplant-induced I/R injury (Ban et al., 2020). In addition, findings in our lab show that Wnt/β-catenin signaling is inhibited in Dox-induced cardiotoxicity; DKK1 and extracellular sFRP1 exacerbates Dox-induced cardiac injury by inhibiting Wnt/β-catenin signaling (Hu et al., 2019; Liang et al., 2019). Therefore, targeting Wnt/β-catenin signaling may effectively ameliorate Dox-induced cardiotoxicity.

This study explored the role of PKC-ζ in Dox-induced cardiotoxicity and underlying mechanisms. Results of the current study indicated that PKC-ζ acted as upstream suppressor of Wnt/β-catenin signaling and aggravated Dox-induced cardiac injury. PKC-ζ-based intervention was a potential strategy for alleviating Dox-induced cardiotoxicity.

## Materials and Methods

### Reagents

Dox (purity 99.36%) was purchased from Target Molecule Corp (United States). Small-interfering RNA (siRNA) of PKC-ζ was purchased from Genema (Shanghai, China). The PKC-ζ pseudo-substrate inhibitor Myristoylated was purchased from Santa Cruz (United States). XAV-939 was purchased from Selleck Chemicals (United States). LiCl was purchased from Sigma (United States).

### Animals

Animal experimental procedures were undertaken in compliance with the *Guide for the Care and Use of Laboratory Animals* (NIH Publication No. 85–23, revised 1996) and approved by the Research Ethics Committee of Sun Yat-sen University. Male Sprague–Dawley (SD) rats (weighing 220–250 g, SPF grade, certification No. 44008500019766) were purchased from the Experimental Animal Center of Sun Yat-sen University (Guangzhou, China). Animals were randomly divided into two groups, model and control groups, and each group contained 6 rats. Rats in the model group were intraperitoneally administered with Dox on the 1st, 5th, and 9th day at a dosage of 5 mg/kg. Rats in the control group were intraperitoneally administered with the same dosage of normal saline (NS).

### Echocardiographic and Morphometric Measurements

The day after the final administration of Dox, SD rats were anesthetized with 3% isoflurane, and echocardiographic measurements were taken using Technos MPX ultrasound system (ESAOTE, Italy) of two-dimensional-guided M-mode echocardiography based on previous studies (Li et al., 2019). Cardiac indices including ejection fraction (EF), fractional shortening (FS), left ventricular end-systolic posterior wall thickness (LVPWs), and left ventricular end-diastolic posterior wall thickness (LVPWd) were determined. After finishing echocardiographic measurements, rats were immediately sacrificed and their heart tissues were removed after injected with 0.1 M KCl. In order to conduct morphometric measurement, hearts were transversely trimmed into two parts, one part was cut into 5-μm-thick histological cross sections and fixed in 4% paraformaldehyde, which were used for hematoxylin–eosin (HE), Picrosirius Red (PSR), and TUNEL staining. The other part of heart tissues was stored in −80°C, which was used for immunoblotting and mRNA detection.

### Cell Culture

Heart tissues of SD rats (1–3 days old) were isolated based on a previously described protocol (Feng et al., 2015) to obtain neonatal rat cardiomyocytes (NRCMs). NRCMs were then incubated in Dulbecco’s modified Eagle’s medium (DMEM) with 10% fetal bovine serum (FBS) and 0.1 mM 5-bromodeoxyuridine for 24 h. NRCMs were then washed with PBS and cultured in new DMEM supplemented with 10% FBS for 12 h before treatment. NRCMs were treated with Dox for 12 h to induce cardiomyocyte injury.

### Immunoblotting

Proteins of cardiomyocytes or heart tissues were extracted using RIPA lysis buffer. Equal amounts of 30- or 35-μg proteins were used to undertake immunoblotting experiment as previously described (Huang et al., 2017). Primary antibodies against cleaved-caspase3 (diluted 1:1,000), PARP1 (diluted 1:1,000), and p-PKC-ζ (Thr410) (diluted 1:500) were purchased from Cell Signaling Technology. Primary antibodies against Bax (diluted 1:1,000) were purchased from Abcam. In addition, primary antibodies against α-tubulin (diluted 1:5,000), caspase3 (diluted 1:1,000), and β-catenin (diluted 1:1,000) were purchased from Proteintech Group. Primary antibodies against Bcl-2 were purchased from Boster (diluted 1:500). Finally, primary antibodies against PKC-ζ were purchased from Santa Cruz (diluted 1:500).

### Transfections of Plasmids and Small-Interfering RNA

DNA sequence of pEGFP-N3-Flag-PKC-ζ plasmid was confirmed by Sangon Biotech Co. Ltd. (Shanghai). Plasmid was transiently transfected into cardiomyocytes based on the manufacturer’s instructions of Lipo 2000. The siRNA sequence of PKC-ζ included sense 5′-GCA​AGC​UGC​UUG​UCC​AUA​ATT-3′ and antisense 5′-UUA​UGG​ACA​AGC​AGC​UUG​CTT-3’. Cardiomyocytes were transfected with siRNA or negative control based on RNAiMAX transfection reagent instructions for use. All experiments were conducted in 48–72 h after transfection.

### Detection of Nuclear Condensation, Mitochondrial Membrane Potential, and Mitochondrial Morphology

Cardiomyocytes were seeded into 48-well plates. After corresponding treatments, cells were washed thrice with PBS and then incubated with 10 nM tetra-methylrhodamine ethyl ester (TMRE) (Invitrogen, United States) for 30 min followed by 10 μg/ml Hoechst 33,42 staining (Solarbio, China) for 10 min at 37°C. Mitochondrial membrane potential and nuclear condensation were detected using EVOS FL Auto (Life Technologies, Bothell, WA, United States).

After washing with PBS, cardiomyocytes were fixed with 4% paraformaldehyde for 15 min and incubated with 0.3% Triton X-100 for 10 min at room temperature; cardiomyocytes were then incubated with 1 μM MitoTracker Red (Invitrogen, United States) for 30 min followed by 10 μg/ml Hoechst 33342 staining for 10 min. Mitochondrial morphology was detected using an ultra-high-resolution laser scanning microscope (Olympus, Japan).

### Immunofluorescence

Cardiomyocytes were seeded into coverglass bottom dishes. After washing thrice with PBS, cells were fixed with 4% paraformaldehyde (20 min) and permeabilized using 0.3% Triton X-100 (10 min). Then, cells were washed with PBS for another three times and were blocked with 10% goat serum at room temperature for 1 h. Antibodies against PKC-ζ (diluted 1:50), β-catenin (diluted 1:100), *p*-PKC-ζ (Santa Cruz, diluted 1:100), or Troponin T (Santa Cruz, diluted 1:100) were used to incubate cells overnight at 4°C. Next, cardiomyocytes were incubated with Alexa Fluor 488- or 594-conjugated anti-rabbit or mouse IgG (H + L) secondary antibody (Proteintech Group, diluted 1:200) at room temperature for 1 h. After nuclear staining by DAPI for 10 min, cardiomyocytes were detected using an ultra-high-resolution laser scanning microscope (Olympus, Japan).

### Co-Immunoprecipitation

A total of 300–400 μg of protein was respectively incubated with anti-β-catenin (diluted 1:100) or anti-PKC-ζ antibody (diluted 1:50) and IgG antibody (Beyotime, China) overnight at 4°C, followed by incubation with 20 μl of protein A/G beads (Pierce, United States) for 4 h at 4°C. Then, beads were washed with IP Lysis buffer and diluted in loading buffer. The interactive protein was assayed by immunoblotting.

### Statistical Analyses

Data are presented as mean ± SEM. Statistical analyses between two groups were done using Student’s *t*-tests. One-way ANOVA with Bonferroni post hoc tests were undertaken for multiple group comparisons. *p* < 0.05 was considered statistically significant.

## Results

### PKC-ζ Phosphorylation Was Increased by Dox Treatment

**FIGURE 1:**
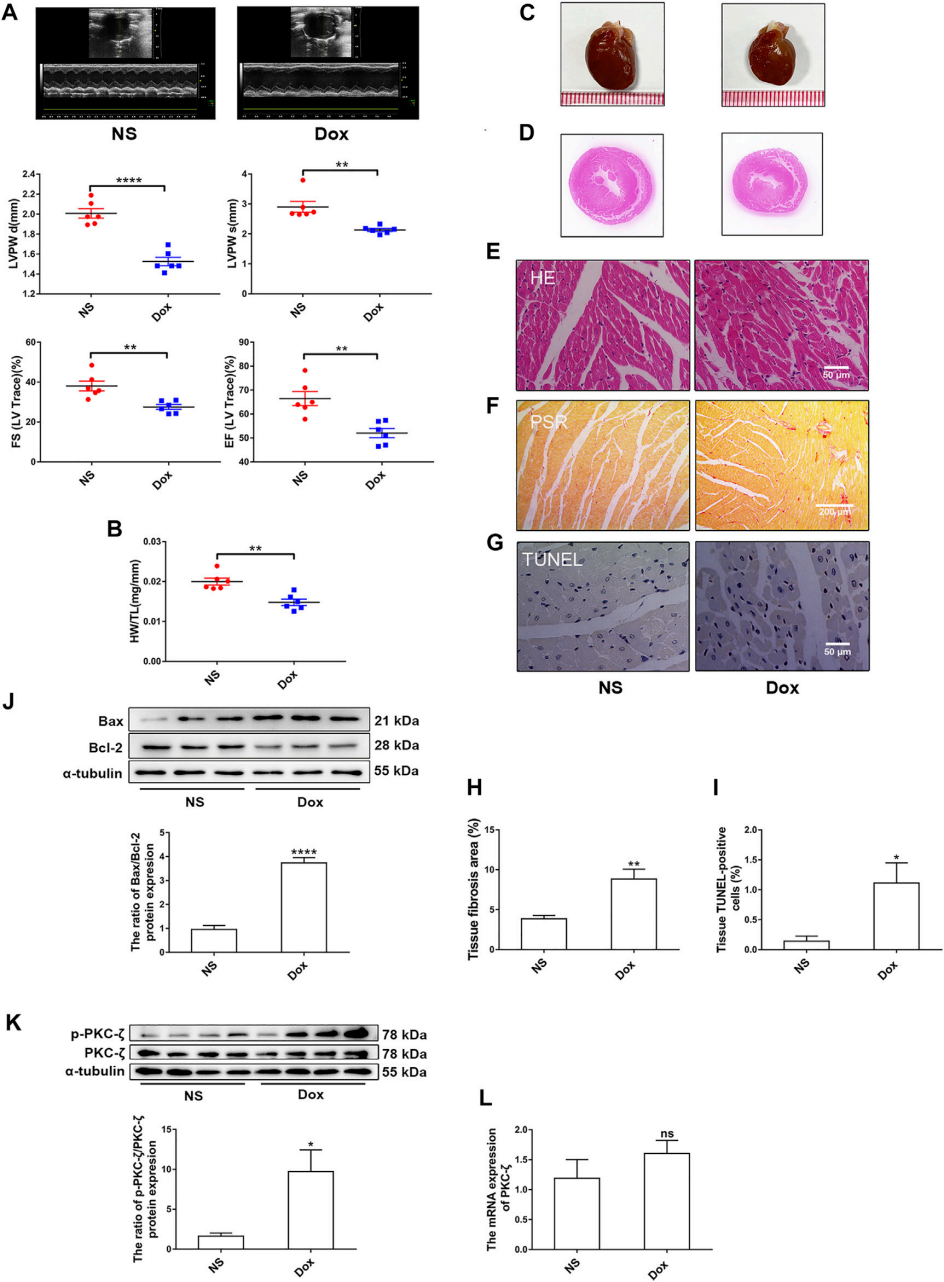
PKC-ζ phosphorylation was upregulated by Dox treatment *in vivo*. Sprague–Dawley (SD) rats were intraperitoneally administered with Dox with cumulative dose of 15 mg/kg body weight to induce cardiotoxicity. Rats in the control group were given the same dosage of normal saline (NS), *n* = 6. **(A)** Ejection fraction (EF%), fractional shortening (FS%), and left ventricular posterior wall thickness (LVPW) were detected by echocardiographic graphs. **(B)** Changes of heart weight-to-tibia length (HW/TL) ratio were determined. **(C, D)** Changes of heart sizes were detected. **(E)** HE staining of left ventricle. Scale bars: 50 μm. **(F, H)** Cardiac fibrosis area was determined by PSR staining. (Scale bars: 200 μm, *n* = 4). **(G, I)** Cardiac apoptosis was determined by TUNEL staining. (Scale bars: 50 μm, *n* = 4). **(J)** Relative protein level of Bax to Bcl-2 was detected by Western blot (*n* = 6). **(K)** Phosphorylation level of PKC-ζ was determined using Western blot (*n* = 4). (**L**) PKC-ζ mRNA level was detected by qRT-PCR (*n* = 6). Data are presented as means ± SEM. *p < 0.05, **p < 0.01 vs. control group.

To explore the role of PKC-ζ in Dox-induced cardiac injury, we examined the change of PKC-ζ phosphorylation level *in vivo* and *in vitro*. SD rats were intraperitoneally administered with Dox at a dosage of 5 mg/kg body weight on the 1st, 5th, and 9th day, leading to a cumulative dosage of 15 mg/kg body weight. The echocardiographic analysis revealed decreased ejection fraction (EF%), fractional shortening (FS%), and left ventricular posterior wall thickness (LVPW) in Dox-treated group rats compared with those of control group rats (Figure 1A). Heart weight-to-tibia length ratio was decreased in comparison with those of control group rats (Figure 1B). Figures 1C,D showed that hearts of rats in the Dox-treated group were smaller. HE staining as well as PSR staining indicated aggravation of cardiomyocyte disorganization and cardiac fibrosis, respectively (Figures 1E,F,H). Moreover, TUNEL staining and protein level of Bax/Bcl-2 revealed some degree of elevated apoptosis in the Dox group (Figures 1G,I,J). As indicated in Figures 1K,L, administration of Dox increased Thr410 phosphorylation of PKC-ζ, although it had no effect on total protein and mRNA levels of PKC-ζ.

**FIGURE 2:**
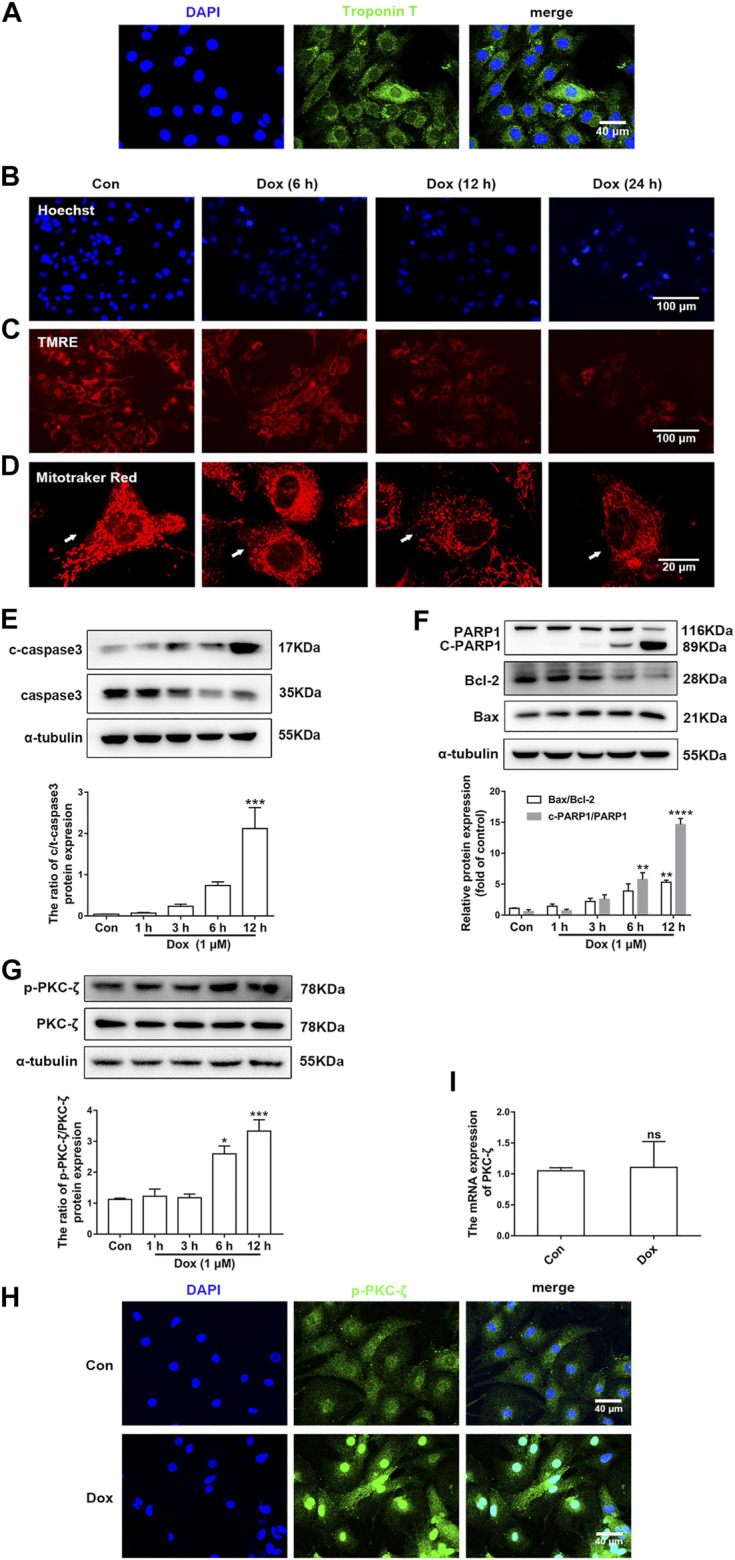
PKC-ζ phosphorylation was upregulated by Dox treatment *in vitro*. **(A)** The expression of Troponin T in NRCMs was detected by IF. Scale bar: 40 μm. **(B)** Cardiomyocytes were incubated with 1 µM Dox for the indicated time; Hoechst 33342 staining was used to detect nuclear condensation. Scale bar: 100 μm. **(C)** TMRE staining revealed mitochondrial membrane potential change. Scale bar: 100 μm. **(D)** MitoTracker Red staining was applied to detect mitochondria morphology. Scale bar: 20 μm. **(E, F)** c-PARP1/PARP1, Bax/Bcl-2, and c-caspase3/caspase3 ratios were detected by Western blot. **(G)** Phosphorylation and total protein level of PKC-ζ were detected by Western blot. **(H)** Phosphorylation level of PKC-ζ by Dox stimulation for 12 h was detected by immunofluorescence. **(I)** qRT-PCR showed mRNA level of PKC-ζ. Data were presented as means ± SEM. *p < 0.05, **p < 0.01 vs. control group, *n* = 3.

Figure 2A indicated that the purity of cardiomyocytes isolated from NRCMs was more than 95%, which was observed by IF staining of Troponin T. Next, cardiomyocytes were then treated with 1 μM Dox at different times and the change of PKC-ζ was explored *in vitro*. It was found that Dox treatment led to nuclear condensation, decreased mitochondrial membrane potential, and mitochondrial morphological disorder (Figures 2B–D, Supplementary Figure S1A). In addition, Dox caused a time-dependent increase in apoptosis indices including cleaved-PARP1/PARP1, Bax/Bcl-2, and cleaved-caspase3/caspase3 (Figures 2E,F). Based on these results, cardiomyocytes were stimulated with 1 μM Dox for 12 h to induce cardiotoxicity in subsequent experiments. Notably, results of *in vitro* levels of Thr410 phosphorylation of PKC-ζ as well as total protein and mRNA levels of PKC-ζ were parallel to findings generated *in vivo* (Figures 2G–I). These results, therefore, indicated that Dox treatment increased PKC-ζ phosphorylation level and induced PKC-ζ activation.

### PKC-ζ Overexpression Aggravated Dox-Induced Cardiotoxicity

**FIGURE 3:**
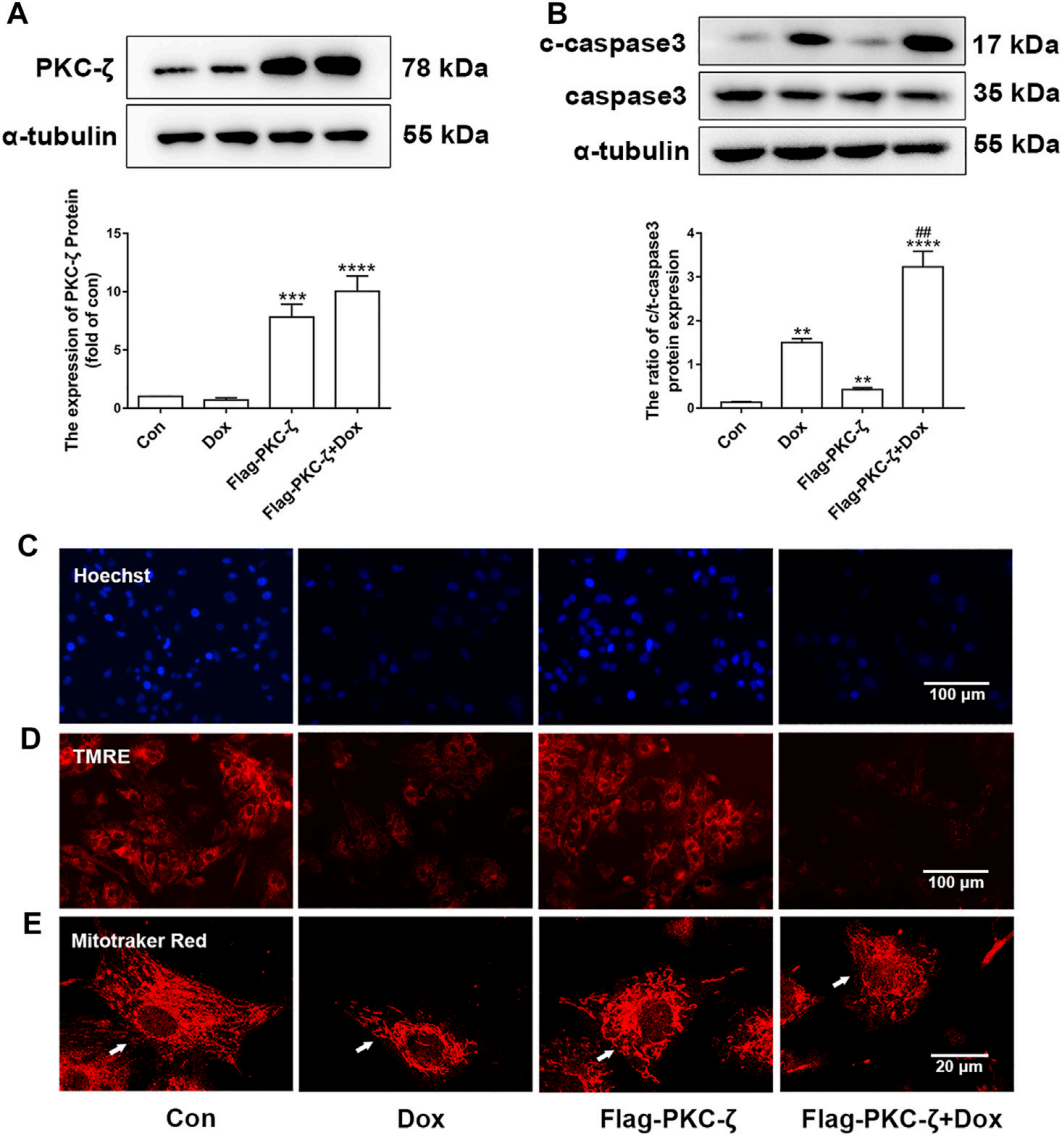
PKC-ζ overexpression exacerbated Dox-induced cardiac injury. **(A)** Cardiomyocytes were transfected with Flag-PKC-ζ plasmid and treated with 1 µM Dox for 12 h; protein level of PKC-ζ was determined by Western blot. **(B)** Ratio of c-caspase3/caspase3 was determined by Western blot. **(C–E)** Nuclear condensation, mitochondrial membrane potential (Δψm), and morphology of mitochondria were determined by Hoechst 33342 (scale bar: 100 μm), TMRE (scale bar: 100 μm), and MitoTracker Red staining (scale bar: 20 μm), respectively. Data were presented as means ± SEM. **p < 0.01 vs. control group; ^##^ *p* < 0.01 vs. Dox group, *n* = 3.

The role of PKC-ζ in Dox-induced cardiac injury was determined by transfecting Flag-PKC-ζ plasmid into cardiomyocytes. PKC-ζ was successfully overexpressed as shown in Figure 3A. Results shown in Figure 3B indicated that PKC-ζ overexpression exacerbated Dox-induced apoptosis, which was reflected by increased cleaved-caspase3/caspase3 ratio. Moreover, PKC-ζ overexpression exacerbated nuclear condensation, mitochondrial membrane reduction, and mitochondrial morphology disorder caused by Dox (Figures 3C–E, Supplementary Figure S1B). These findings indicated that PKC-ζ overexpression aggravated Dox-induced cardiotoxicity.

### PKC-ζ Knockdown or Inhibition Attenuated Dox-Induced Cardiotoxicity

**FIGURE 4:**
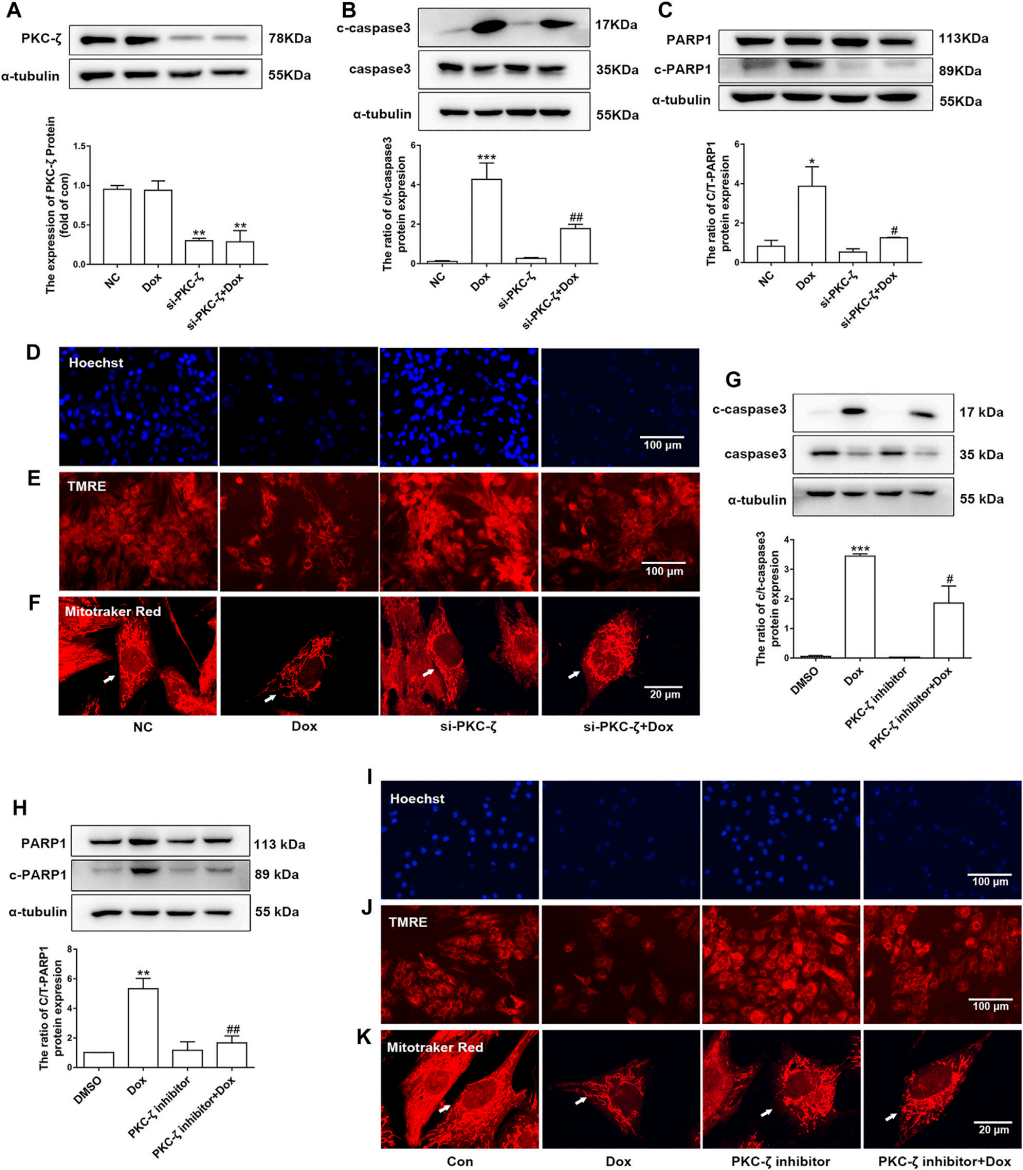
Knockdown and inhibition of PKC-ζ attenuated Dox-induced cardiac injury. **(A)** Cardiomyocytes were transfected with PKC-ζ siRNA and treated with Dox (1 μM, 12 h); interference efficiency was detected by Western blot. **(B, C)** C-caspase3-to-caspase3 and c-PARP1-to-PARP1 ratios were determined by Western blot. **(D–F)** Nuclear condensation, mitochondrial membrane potential (Δψm), and morphology of mitochondria were determined by Hoechst 33342 (scale bar: 100 μm), TMRE (scale bar: 100 μm), and MitoTracker Red staining (scale bar: 20 μm), respectively. **(G, H)** Cardiomyocytes were incubated with PKC-ζ pseudo-substrate inhibitor (10 µM) for 1 h before Dox treatment (1 μM, 12 h). Ratios of c-caspase3/caspase3 and c-PARP1/PARP1 were detected by Western blot. **(I–K)** Nuclear condensation, mitochondria membrane potential, and mitochondrial morphology were determined by staining with Hoechst 33342 (scale bar: 100 μm), TMRE (scale bar: 100 μm), and MitoTracker Red staining (scale bar: 20 μm), respectively. Data were presented as means ± SEM. *p < 0.05, **p < 0.01 vs. control group. ^#^ *p* < 0.05, ^##^ *p* < 0.01 vs. Dox group, *n* = 3.

PKC-ζ siRNA were used to knockdown PKC-ζ. Knockdown efficiency of PKC-ζ siRNA was confirmed in Figure 4A. As shown in Figures 4B,C, PKC-ζ knockdown attenuated Dox-induced apoptosis of cardiomyocytes, as shown by inhibiting the cleavage of caspase3 and PARP1. In addition, PKC-ζ knockdown attenuated mitochondrial membrane depolarization, nuclear condensation, and mitochondrial morphology disorder due to Dox treatment (Figures 4D–F, Supplementary Figure S1C).

Similarly, the PKC-ζ pseudo-substrate inhibitor Myristoylated blocked Dox-induced increases in cleaved-PARP1/PARP1 and cleaved-caspase3/caspase3 ratio, as well as mitochondrial dysfunction (Figures 4G–K), suggesting that inhibition of PKC-ζ activity attenuated Dox-induced cardiotoxicity.

These results revealed that PKC-ζ was an essential regulatory factor in Dox-induced cardiac injury.

### PKC-ζ Interacted With β-Catenin and Inhibited Wnt/β-Catenin Signaling Pathway

**FIGURE 5:**
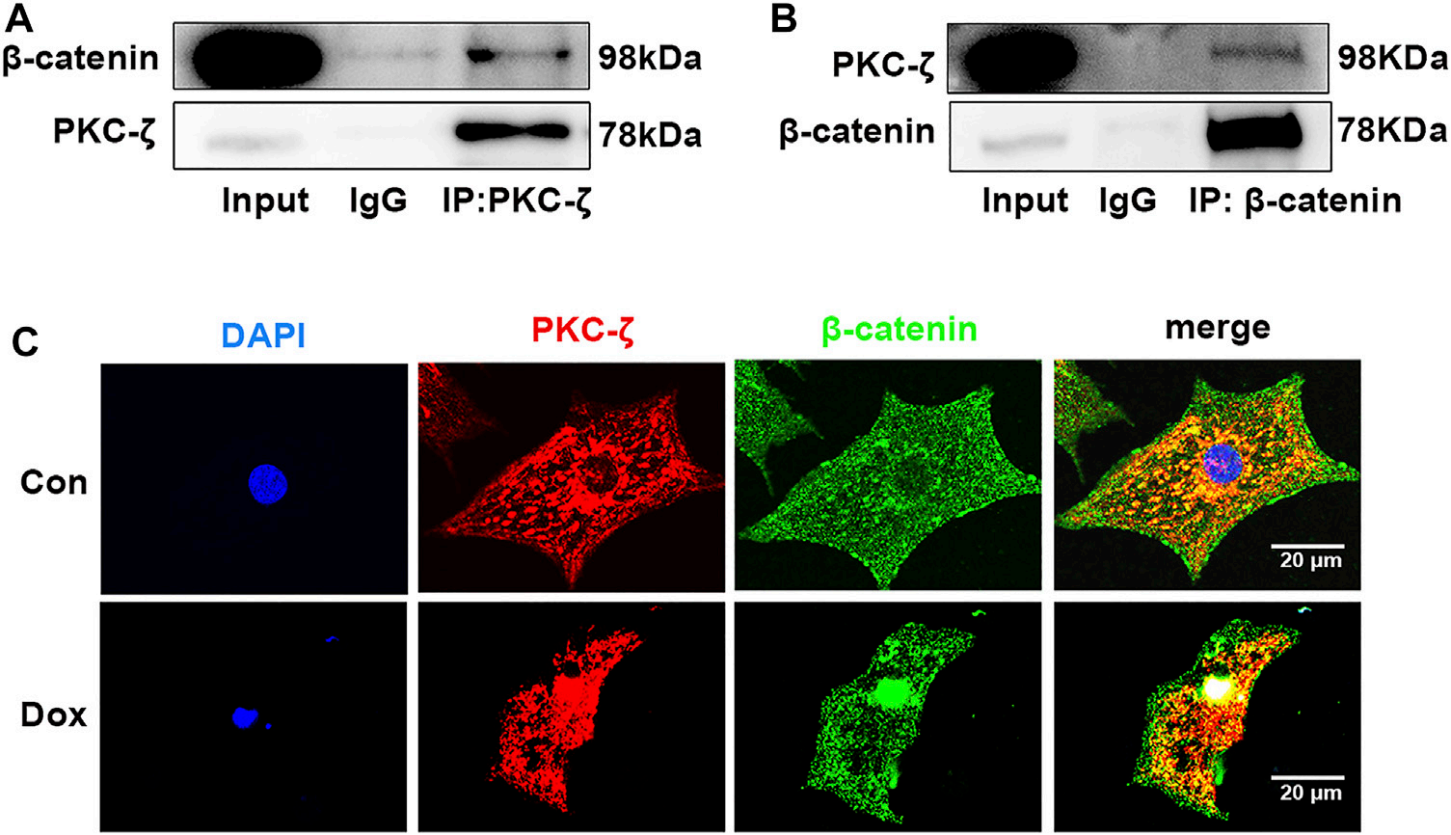
PKC-ζ co-localized and interacted with β-catenin. **(A, B)** Co-IP was undertaken to explore interaction between PKC-ζ and β-catenin. Total protein extracted from cardiomyocytes was immunoprecipitated with anti-PKC-ζ, anti-β-catenin, or anti-IgG antibodies, and interaction of PKC-ζ and β-catenin was detected by Western blot. **(C)** Intracellular co-localization of β-catenin (green) and PKC-ζ (red) were identified by confocal immunofluorescence microscopy. Scale bar: 20 μm.

Potential molecular mechanisms underlying PKC-ζ aggravated Dox-induced cardiotoxicity were then explored. Findings of co-immunoprecipitation analysis showed that PKC-ζ interacted with β-catenin (Figures 5A,B). Confocal immunofluorescence microscopy experiment indicated co-localization of PKC-ζ and β-catenin (Figure 5C).

**FIGURE 6:**
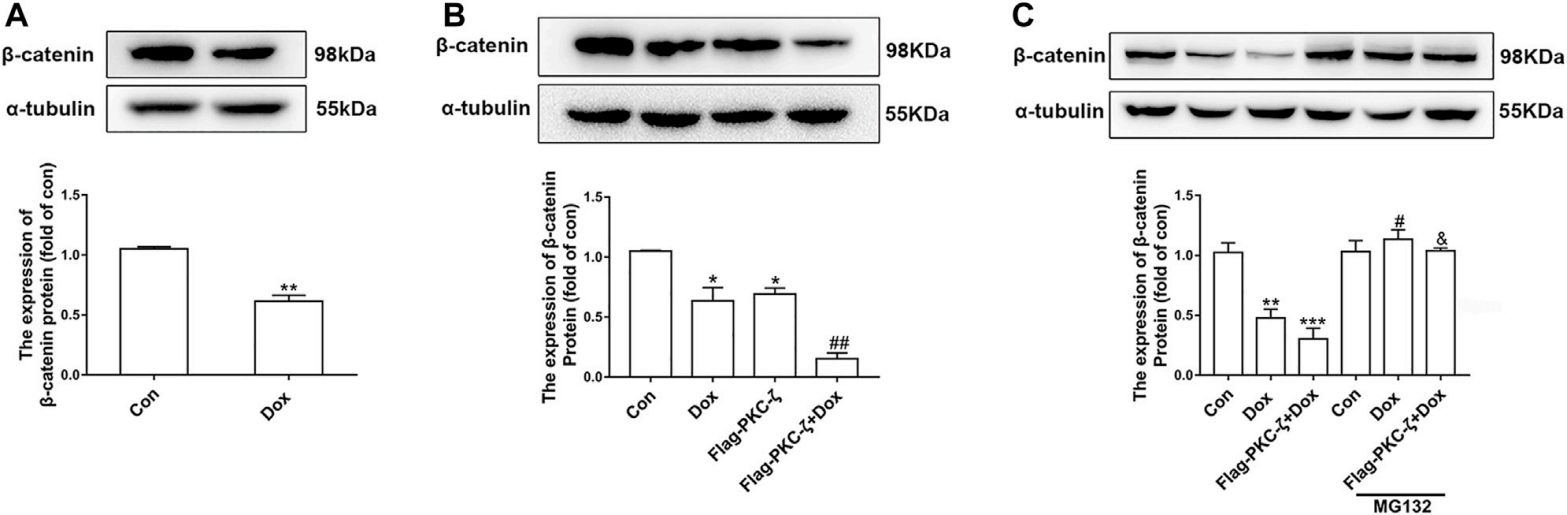
PKC-ζ overexpression inhibited Wnt/β-catenin signaling. **(A)** Change of β-catenin protein level by Dox (1 μM, 12 h) treatment was detected by Western blot. **(B)** Cardiomyocytes were transfected with Flag-PKC-ζ with or without Dox stimulation. β-catenin protein level was determined by Western blot. **(C)** Protein level of β-catenin treated with MG132 was determined by Western blot. Data were presented as means ± SEM. *p < 0.05, **p < 0.01 vs. control group; ^#^ *p* < 0.05, ^##^ *p* < 0.01 vs. Dox group; ^&^ *p* < 0.05 vs. Flag-PKC-ζ+Dox group. *n* = 3.

Then, the regulatory effect of PKC-ζ on Wnt/β-catenin in Dox-induced cardiotoxicity was explored by determination of β-catenin protein level caused by PKC-ζ overexpression and Dox treatment. Dox stimulation decreased β-catenin and cyclin D1 protein level (Figure 6A, Supplementary Figure S2A). PKC-ζ overexpression further inhibited β-catenin when cardiomyocytes were treated with Dox (Figure 6B). However, treatment of cardiomyocytes with MG132 (a proteasome inhibitor, 10 μM, 6 h) blocked the decrease of β-catenin, which was caused by Dox and PKC-ζ (Figure 6C). Therefore, inhibition of Wnt/β-catenin explained PKC-ζ-driven aggravation of Dox-induced cardiotoxicity to some extent.

### Wnt/β-Catenin Signaling Pathway Involved PKC-ζ-Driven Aggravation of Dox-Induced Cardiotoxicity

**FIGURE 7:**
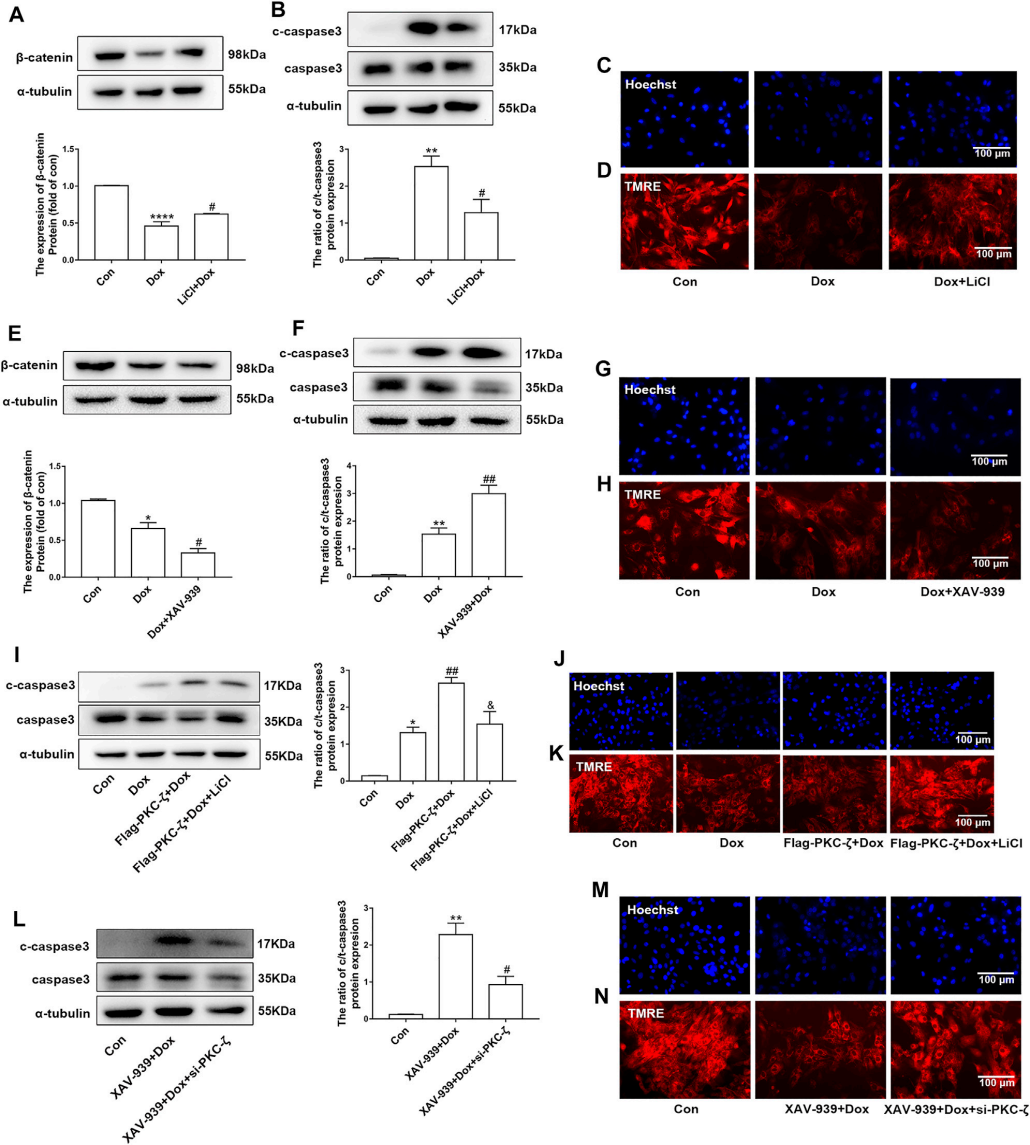
Involvement of Wnt/β-catenin in PKC-ζ-induced Dox cardiotoxicity. **(A)** Cardiomyocytes were incubated with LiCl (5 mM) for 12 h before Dox treatment (1 μM, 12 h). β-catenin protein level was determined by Western blot. **(B)** Apoptosis index c-caspase3/caspase3 was determined by Western blot. **(C, D)** Nuclear condensation and mitochondrial membrane potential were determined by Hoechst 33342 and TMRE staining, respectively. Scale bar: 100 μm. **(E)** Cardiomyocytes were incubated with XAV-939 (5 µM) for 12 h before Dox treatment (1 μM, 12 h). β-catenin protein level was determined by Western blot. **(F)** Apoptosis index (c-caspase3/caspase3 ratio) was determined by Western blot. **(G, H)** Nuclear condensation and mitochondria membrane potential were identified by Hoechst 33342 and TMRE staining, respectively. Scale bar: 100 μm. **(I)** Cardiomyocytes were transfected with Flag-PKC-ζ and then incubated with LiCl and Dox. Western blot analysis was used to detect c-caspase3/caspase3 ratio. **(J, K)** Nuclear condensation and mitochondrial membrane potential were determined by Hoechst 33342 and TMRE staining, respectively. Scale bar: 100 μm. **(L)** Cardiomyocytes were transfected with si-PKC-ζ and then incubated with XAV-939 and Dox. Western blot analysis showed c-caspase3/caspase3 ratio. **(M, N)** Nuclear condensation and mitochondrial membrane potential were determined by Hoechst 33342 and TMRE staining, respectively. Scale bar: 100 μm. Data were presented as means ± SEM. *p < 0.05, **p < 0.01 vs. control group; ^#^ *p* < 0.05, ^##^ *p* < 0.01 vs. Dox group or XAV-939 + Dox group; and *p* < 0.05 vs. Flag-PKC-ζ+Dox group, *n* = 3.

The current study has shown that Wnt/β-catenin signaling was inhibited by PKC-ζ overexpression or Dox treatment (Figures 6A,B). Then, the role of Wnt/β-catenin signaling in Dox-induced cardiotoxicity was detected. Cardiomyocytes were pretreated with 5 mM LiCl (β-catenin activator) or 5 μM XAV-939 (Wnt/β-catenin inhibitor) for 12 h followed by Dox treatment. As shown in Figure 7A, LiCl upregulated β-catenin protein level when treated with Dox. LiCl attenuated Dox-induced apoptosis of cardiomyocytes, nuclear condensation, and decline of mitochondrial membrane potential (Figures 7B–D). However, pretreatment with XAV-939 further decreased β-catenin protein level caused by Dox (Figure 7E). XAV-939 exerted contrasting effects compared with those of LiCl pretreatment (Figures 7F–H), an indication that activation of Wnt/β-catenin signaling protected against Dox-induced cardiotoxicity.

Then, we explored the role of Wnt/β-catenin in PKC-ζ-driven Dox-induced cardiotoxicity by simultaneously intervening Wnt/β-catenin and PKC-ζ. Results showed that LiCl alleviated PKC-ζ overexpression-induced cardiomyocytes apoptosis, nuclear condensation, and decline of mitochondrial membrane potential (Figures 7I–K), whereas knockdown of PKC-ζ alleviated XAV939-induced apoptosis, nuclear condensation, and decline of mitochondrial membrane potential (Figure 7L–N). These findings suggest that activation of Wnt/β-catenin suppressed aggravation of Dox-induced cardiotoxicity due to PKC-ζ overexpression.

## Discussion

Dox is a widely prescribed chemotherapeutic drug, which treats cancer by intercalating with DNA and inhibiting topoisomerase Ⅱ (Renu et al., 2018). However, cardiotoxicity of Dox is among the severe side effects in clinical application. Although several research efforts have explored pathogenesis of Dox-induced cardiotoxicity, its exact mechanism is still obscure. Therefore, more studies are needed to help to develop preventive and cardioprotective drugs. The current study successfully replicated the Dox-induced cardiotoxicity model both *in vitro* and *in vivo*. Our results showed that Dox treatment caused cardiomyocyte apoptosis, nuclear condensation, decline of mitochondrial membrane potential, and mitochondrial morphology disorder. Notably, Dox-treated rats showed decreased left ventricular function and increased cardiac injury.

PKCs are serine/threonine kinases, which are divided into three subfamilies based on their activation mechanisms: classical PKC isoforms (α, βI, βII, and γ), which are regulated by calcium, DAG, and phospholipids; novel PKC isoforms (δ, ε, η, and θ), which are activated by DAG and phospholipids; and atypical PKC isoforms (ζ and λ or ι), whose activation depends on protein–protein interaction and lipids such as ceramide rather than calcium and DAG (Steinberg, 2008). Findings of previous studies confirm that the PKC family played key roles in diseases such as cancer (Reina-Campos et al., 2019), heart failure (Singh et al., 2017), and Alzheimer’s disease (de Barry et al., 2010). PKC-ζ belongs to an atypical PKC subfamily and is considered an important regulatory factor in cardiovascular diseases. For example, oleanonic acid inhibits PKC-ζ-induced nuclear factor-kappa B (NF-κB) activation and protects against phenylephrine (PE)-induced cardiac hypertrophy (Gao et al., 2018). PKC-ζ activity is also inhibited by PICOT and increases cardiac contractility (Oh et al., 2012). Our previous findings established that Sirtuin1 negatively regulated PKC-ζ phosphorylation level and protected cardiomyocytes from PKC-ζ-induced hypertrophy (Li et al., 2019). However, function of PKC-ζ in Dox-induced cardiotoxicity has not been explored. The current study showed that Dox treatment upregulated PKC-ζ Thr410 phosphorylation, and we speculated that this may partly be due to the ceramide generation induced by Dox (Delpy et al., 1999). Then, we found that overexpression of PKC-ζ aggravated Dox-induced cardiotoxicity, which was reflected by decreased mitochondrial membrane potential, elevated level of apoptosis of cardiomyocytes, and nuclear condensation. Conversely, knockdown or inhibition of PKC-ζ showed alleviated cardiomyocyte injury following Dox treatment.

Evidence has shown that the Wnt/β-catenin pathway is implicated in cardiac differentiation (Li et al., 2012), apoptosis, angiogenesis (Reis and Liebner, 2013), hypertrophy (Zhao et al., 2019), and other cardiovascular diseases. For instance, induced pluripotent stem cell-derived conditioned medium (iPS-CM) protects H_2_O_2_-induced H9C2 cells against apoptosis by upregulating the Wnt/β-catenin pathway (Guo et al., 2019). MiR-148b aggravates myocardial I/R injury *via* inhibiting Wnt/β-catenin (Yang et al., 2019). β-catenin has been shown to cause increased eNOS activity and pro-survival effects in TNFα- or H_2_O_2_-induced apoptosis in endothelial cells (Tajadura et al., 2020). Wnt/β‐catenin signaling also plays a protective role in Dox-induced cardiotoxicity. For example, overexpression of lncRNA AC061961.2 activates Wnt/β‐catenin and inhibits cardiomyocyte apoptosis caused by Dox (Qiu et al., 2020). In addition, our lab previously revealed that the β-catenin protein level was decreased by Dox stimulation *in vivo* and *in vitro.* Activating Wnt/β-catenin signaling by LiCl alleviated Dox-induced cardiomyopathy (Hu et al., 2019), while KYA1797K (an inhibitor of β-catenin) showed aggravating cardiotoxicity that was caused by Dox (Liang et al., 2019). Wnt/β‐catenin signaling may be an effective target to relieve cardiotoxicity resulting from Dox. In the current study, we also found that Dox treatment decreased β-catenin protein level. Notably, LiCl (β-catenin activator) alleviated Dox-induced cardiotoxicity, whereas Dox-induced cardiomyocyte injury was aggravated by pretreatment with XAV-939 (Wnt/β-catenin signaling inhibitor). Studies showed that casein kinase 1 (CK1) and APC (adenomatous polyposis coli)/Axin/glycogen synthase kinase-3β (GSK-3β) complex promotes β-catenin degradation through the ubiquitin–proteasome pathway, a process that requires phosphorylation of serine residues (Ser33/Ser37/Ser45) in β-catenin protein (Niehrs, 2012). Several previous studies have established the relationship between PKCs and β-catenin. For example, PKC-α phosphorylated and negatively regulated β-catenin protein level (Gwak et al., 2006). Victoria Llado et al. established that PKC-ζ interacted with β-catenin and downregulated its protein level by phosphorylating Ser45 of β-catenin (Llado et al., 2015). We postulated that Wnt/β-catenin signaling may play a role in aggravation of PKC-ζ-driven Dox-induced cardiac injury. Findings of the current study established that PKC-ζ directly interacted with β-catenin. β-catenin protein level inhibited by Dox treatment was further decreased by PKC-ζ overexpression, and this decline was blocked by treatment with a proteasome inhibitor MG132. In addition, pretreatment with XAV-939 aggravated Dox-induced apoptosis of cardiomyocytes, which was relieved by si-PKC-ζ. Activation of Wnt/β-catenin signaling by LiCl pretreatment repressed c-caspase3/caspase3 ratio, which is caused by PKC-ζ overexpression.

In summary, the current study established that loss of function of PKC-ζ is a potential therapeutic approach to alleviate Dox-induced cardiac injury. In addition, the current study indicated that the deleterious effect of PKC-ζ is partly elucidated by inhibiting Wnt/β-catenin signaling. Further studies on cardiac-specific overexpression or knockdown of PKC-ζ in animal models are needed to further validate all the observed cellular findings in the current study. Moreover, specific regulatory mechanisms underlying PKC-ζ regulation of β-catenin should be explored further.

## Data Availability

The original contributions presented in the study are included in the article/Supplementary Material. Further inquiries can be directed to the corresponding authors.

## Abbreviations

Dox, doxorubicin; DMEM, Dulbecco's modified Eagle's medium; FBS, fetal bovine serum; HE, hematoxylin–eosin; NRCMs, neonatal rat cardiomyocytes; PKC-ζ, protein kinase C-ζ; PSR, Picrosirius Red; TMRE, tetramethylrhodamine ethyl ester.
