# Supplementary material for: PKC-ζ Aggravates Doxorubicin-Induced Cardiotoxicity by Inhibiting Wnt/β-Catenin Signaling
Source: Front Pharmacol. 2022 Feb 14;13:798436. doi: 10.3389/fphar.2022.798436 (PMC8883055; doi:10.3389/fphar.2022.798436)
Supplement: Supplementary file 1 [file DataSheet1.docx]

**Supplementary material**

**PKC-ζ aggravates doxorubicin-induced [cardiomyopathy](https://pubmed.ncbi.nlm.nih.gov/32213135/) by**

**inhibiting Wnt/β-catenin signaling**

Yan-jun Cao ^a,1^, Jing-yan Li ^a,b,1^, Pan-xia Wang ^a^, Zhi-rong Lin ^a^, Wen-jing Yu ^a^, Ji-guo Zhang ^c,^*, Jing Lu ^a,^*, Pei-qing Liu ^a,c,^*

^a^ School of Pharmaceutical Sciences, Sun Yat-sen University, Guangzhou 510006, P.R.China

^b^ International Institute for Translational Chinese Medicine, School of Pharmaceutical Science, Guangzhou University of Chinese Medicine, Guangzhou, Guangdong, 510006, China

^c^ School of Pharmaceutical Sciences，Shandong First Medical University & Shandong

Academy of Medical Sciences, Taian，China

^1^ These authors contributed equally to this paper.

* Corresponding author

Department of Pharmacology and Toxicology, School of Pharmaceutical Sciences, Sun Yat-sen University, 132 East Waihuan Road, Guangzhou Higher Education Mega Center, Guangzhou 510006, P.R.China.

1. mail addresses: liupq@mail.sysu.edu.cn (P. Liu); lujing0504@126.com (J. Lu)

School of Pharmaceutical Sciences, Shandong First Medical University & Shandong Academy of Medical Sciences, 619 Chang Cheng Road, Taian, Shandong 271016，P.R. China

1. mail addresses: jgzhang@sdfmu.edu.cn

**
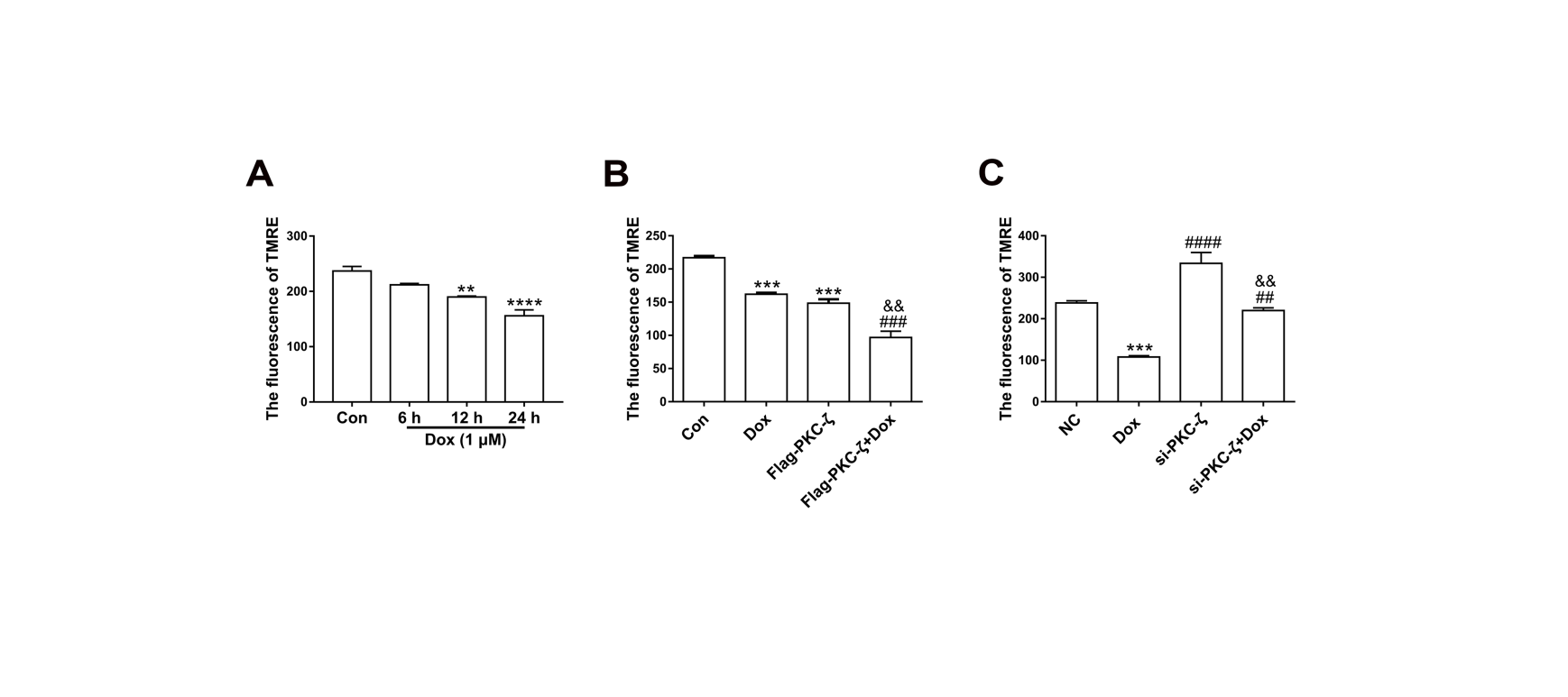
**

**Fig. S1. Effect of PKC-ζ on mitochondrial membrane potential. (A-C)** Cardiomyocytes were treated with Dox for different time point or transfected with Flag-PKC-ζ plasmid and siRNA. Mitochondria membrane potential was measured by Automatic cytome high content screening and analysis system with TMRE staining. ***P* < 0.01 *vs*. the control group, ^#^*P* < 0.05 *vs*. Dox group, ^&^*P* < 0.05 *vs*. Flag-PKC-ζ or si-PKC-ζ group, *n*=3.

**
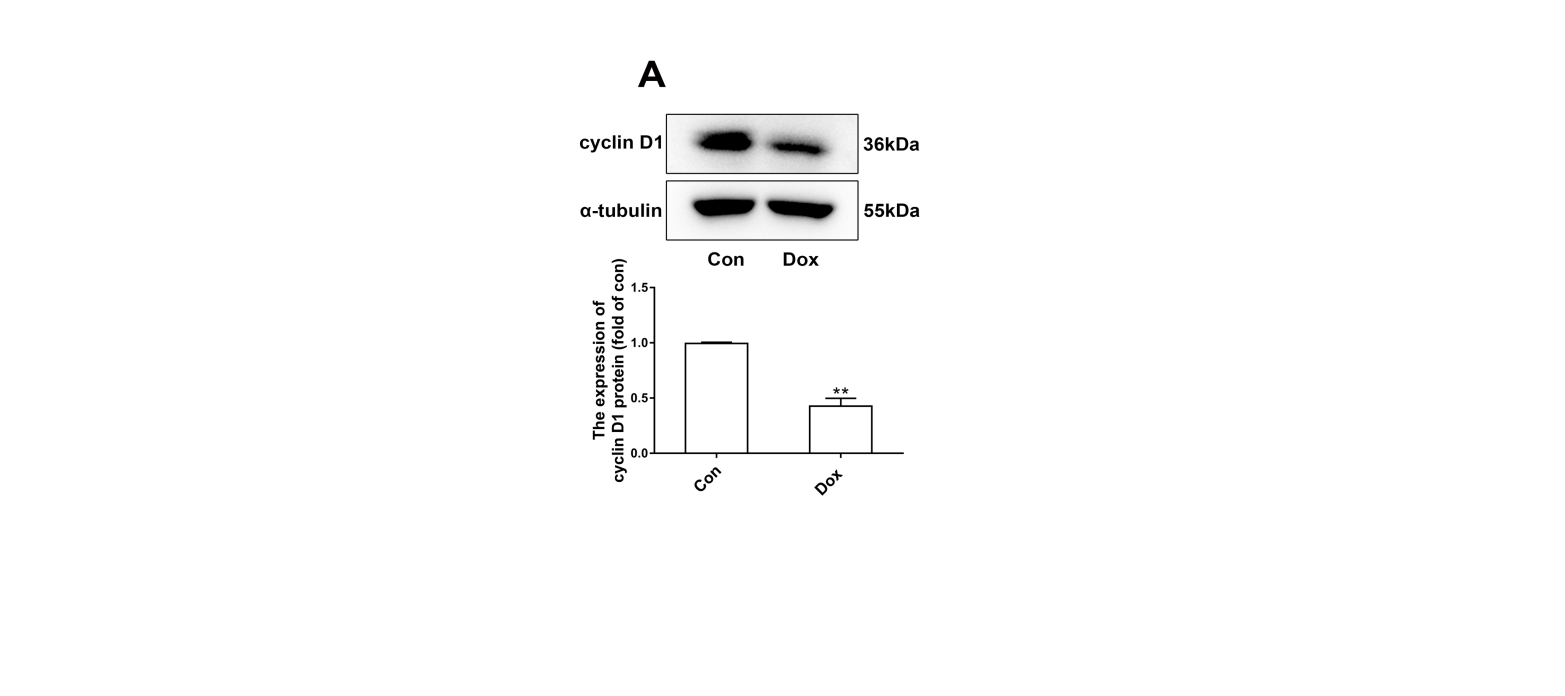
**

**Fig. S2. The change of cyclin D1 by Dox stimulation. (A)** The protein level of cyclin D1 was determined by Western blot. The results were presented as the means ± SEM. ***P* < 0.01 *vs*. the control group, *n*=3.
